# Impact of Social and Relational Adversity on Access to Services among US Children with Autism Spectrum Disorder 2016–2019

**DOI:** 10.3390/children8121099

**Published:** 2021-11-29

**Authors:** Sarah D. Ronis, Eunice Lee, Carrie Cuffman, Kimberly Burkhart

**Affiliations:** 1 Center for Child Health and Policy, UH Rainbow Babies and Children’s Hospital, 11100 Euclid Avenue, MS 6036, Cleveland, OH 44106, USA; 2 Department of Pediatrics, School of Medicine, Case Western Reserve University, 10900 Euclid Avenue, Cleveland, OH 44106, USA; exl182@case.edu (E.L.); kimberly.burkhart@uhhospitals.org (K.B.); 3 Center for Developmental Pediatrics, Cleveland Clinic Children’s, 2801 Martin Luther King Drive, Cleveland, OH 44104, USA; cuffmac2@ccf.org; 4 Division of Developmental and Behavioral Pediatrics and Psychology, UH Rainbow Babies and Children’s Hospital, W.O. Walker Building, 10524 Euclid Avenue Suite 3150, Cleveland, OH 44106, USA

**Keywords:** autism spectrum disorder, adverse childhood experiences, health disparities, social determinants of health, health services

## Abstract

To explore the impact of social and relational adversity on access to key health services among US children with autism spectrum disorders (ASD), cross-sectional analyses of the 2016–2019 National Survey of Children’s Health assessed use of key health services by children with ASD, accounting for differences in demographic characteristics, medical needs, and experience of social and relational adversities. sUS children with ASD were more than twice as likely as peers without ASD to report two or more social adversities and more than three times as likely to report two or more relational adversities. In multivariable models, relational adversities were significantly associated with greater odds of medication use for ASD (OR 1.50, 95%CI:1.02, 2.17). Social adversities were neither associated with receipt of behavioral therapies nor prescription of medication to treat ASD. Screening for various forms of adversity among youth with ASD is of great importance; even one adverse experience may be enough to influence care of a child with ASD, with differences in effect according to the nature of the particular adversity. Further research should evaluate the role that childhood adversity plays in physical and mental health outcomes in ASD.

## 1. Introduction

Social determinants of health are the conditions in which one is born, lives, learns, plays, and works [1]. Adverse childhood experiences (ACEs) comprise a set of consequential social determinants of health and include experiences such as poverty, abuse, neglect, parental mental illness, parental substance use, divorce, incarceration, and exposure to domestic violence [2]. Research has shown that youth with ACEs are more likely to experience a variety of negative outcomes in adulthood including poor physical and mental health, substance abuse, and risky behaviors [2,3]. Nevertheless, a robust emerging literature also conclusively demonstrates that history is not destiny: formation of safe stable nurturing relationships can buffer youth with ACEs against toxic stress [4]. Indeed, a recent policy statement from the American Academy of Pediatrics calls on health care providers to buffer all children against toxic stress through timely provision of both preventive and therapeutic services aimed at promoting relational health [5].

More so than for typical peers, these services may be especially crucial for children and youth with autism spectrum disorder (ASD). Characterized by persistent deficits in social communication and interaction across multiple contexts, ASD is highly prevalent, affecting approximately 1 in 54 U.S. children and youth [6]. Given higher rates of internalizing disorders [7,8], poorer general health [9], and less social connectedness [10,11] as compared to typical peers, US children with ASD may be at increased risk for negative consequences of adverse experiences. Reduced coping mechanisms [8], alterations in sleep [12], and other neurological differences may also make youth with ASD particularly vulnerable to the impact of ACEs due to greater challenges in establishing relationships that buffer the effects of toxic stress [4,13]. Adequate and timely use of appropriate health services, including therapies designed to meet their unique social and behavioral needs, may be essential to promote the wellbeing of ACE-exposed children with ASD.

Prior literature has explored the influence of social and relational factors on the timing of autism diagnosis and initiation of therapy, but have been limited by inconsistencies in their characterization of risk. For example, some studies focusing on racial disparities in timeliness of diagnosis have documented delays in care among African American and Latino children as compared to non-Hispanic white children with ASD [14,15], while others have found no such association [16]. In studies focused on family socioeconomic status, higher household income has in some cases been shown to be associated with more timely care [17]. In more recent literature, the presence and number of ACEs reported by a child with ASD has been shown to be associated with delayed entry into services [18]. However, much of this literature relies on data that predate the expansion of access to the patient-centered medical home and adequate health insurance under the Affordable Care Act, is based on small samples, and has been limited in its ability to account for intersectionality among the various adversities (e.g., race vs. experience of racism, and interpersonal relational factors vs. household socioeconomic factors) that together influence children’s access to health care.

Thus, the purpose of the present study was to characterize the relationships among ACEs and access to services for U.S. children with ASD, using a recent large nationally representative dataset (National Survey of Children’s Health (NSCH) 2016–2019) that could overcome the limitations of prior studies. First, we sought to describe the sociodemographic characteristics of US children with ASD and explore their associations with the types of ACEs these children have experienced. Second, following the Anderson and Aday Framework of Health Services Utilization [19,20], we aimed in multivariable analyses to explore whether and to what extent various adversities function as enabling resources influencing the use of health services pertinent to the management of ASD, specifically, prescription medications to treat symptoms of ASD, and enrollment in behavioral therapies.

## 2. Materials and Methods

### 2.1. Data Source

We performed cross-sectional secondary analysis of the National Survey of Children’s Health (NSCH). The NSCH is an annual mailed or web-based survey distributed by the US Census Bureau in all 50 states and the District of Columbia. The survey is completed by the parent or guardian most knowledgeable about the health and health care of a randomly selected child in the household, yielding nationally representative estimates for non-institutionalized US children ages 0–17 years. We used codebooks made available by the Child and Adolescent Health Measurement Initiative (CAHMI) to construct composite indicators, following conventions defined by the US Maternal and Child Health Bureau (MCHB), that describe the number and types of problems faced by the child, parent-perceived illness severity, functional abilities of the child, impact on the family, needs for and use of services, and family social context. The composite indicators were constructed from variables in the publicly available NSCH 2016, 2017, 2018 and 2019 databases. Full details regarding survey design, implementation, and indicator construction are described elsewhere (https://www.census.gov/programs-surveys/nsch/technical-documentation.html; https://www.childhealthdata.org/learn-about-the-nsch/NSCH, accessed 2 August 2021).

### 2.2. Study Sample

For the purpose of this study, the subsample of children of interest included those who had positive responses to the following two questions, “Has a doctor or other healthcare provider EVER told you that this child has Autism or Autism Spectrum Disorder (ASD) including diagnoses of Asperger’s Disorder or Pervasive Developmental Disorder (PDD)?” and “Does this child currently have this condition?”.

### 2.3. Materials and Design

Health outcomes among children with ASD with and without exposure to childhood adversity were compared for two primary endpoints, (1) current use of medications to treat symptoms of ASD, defined in the NSCH as a positive response to the question “Is this child currently taking medication for autism or autism spectrum disorder?” and (2) enrollment in behavioral therapies to treat ASD, defined in the NSCH as a positive response to the question “At any time during the past 12 months, did this child receive behavioral treatment for autism, autism spectrum disorder, Asperger’s Disorder, or pervasive developmental disorder, such as training or an intervention that you or child received to help with his or her behavior?”.

Explanatory variables of interest included children’s exposure to adversities that might modify their families’ capacity to access such services to care for their child with ASD, including for example, household annual income (percentage of federal poverty level of household), caregiver education (less than high school, high-school or GED completed, some post-secondary education, or post-secondary completed), adequacy of health insurance coverage, care that meets criteria for a medical home (usual source of care with a doctor or nurse who knows the child well, care that is family centered, well-coordinated, and assists with needed referrals), and lifetime exposure (yes or no) to each of nine adverse childhood experiences. Following Bethell and colleagues’ conceptualization of “whole child complexity” [21], we organized adversities into two domains: (1) social adversities, defined as food insecurity, household economic hardship, residence in an unsafe neighborhood, and/or child discriminated against based on their race/ethnicity; and (2) relational adversities, defined as a parent in self-described poor or fair mental health, a parent who reports being frequently angry or aggravated with their child, a parent who reports poor coping with the demands of parenting, or a child who has experienced one or more of six of Felliti’s “classic” ACEs (i.e., parental divorce, death or incarceration of a parent, witness to domestic violence, and/or and lives with an adult in the household who has mental illness and/or uses substance) [3].

Child-level covariates of interest included sex (male/female), current age in years, race/ethnicity (non-Hispanic White, non-Hispanic Black, Hispanic, mixed race/other), parent-reported severity of ASD (mild, moderate, or severe), and medical risks, defined as presence of functional limitations, complex special health care needs, fair/poor overall health status, and/or presence of other chronic conditions in addition to their ASD.

### 2.4. Analysis

**Figure 1:**
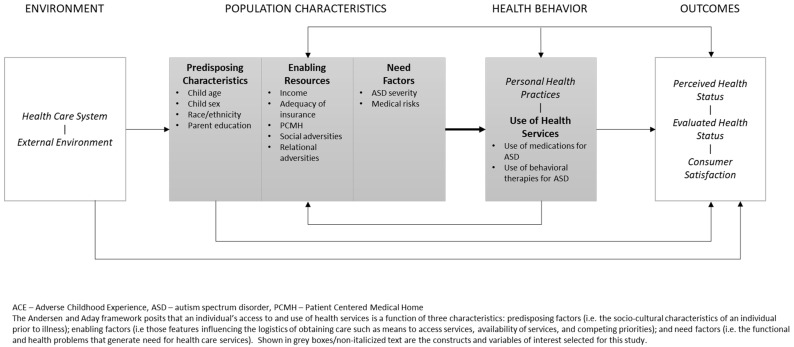
Application of the Andersen and Aday Framework of Health Services Utilization.

Following the Anderson and Aday behavioral model of health services utilization [19,20], we selected the following variables for consideration as potential confounders of the relationship among adversity and ASD-related health service use (see Figure 1): Predisposing characteristics included child age at time of interview, child gender, child race/ethnicity, and highest level of parent educational attainment. Potential enabling resources included household income, adequacy of insurance coverage, and care meeting criteria for patient-centered medical home. Potential need factors included current ASD severity and presence of two or more medical risks.

Univariate frequency distributions were examined for demographics, predictors, and the outcome variables. Independent-samples *t*-tests and chi-square tests were conducted to assess for significant associations among demographics, the different adversities, and the two ASD treatment variables. Those bivariate associations with *p*-value of less than 0.05 were considered as potential confounders for inclusion in multivariable models. 

Multivariate logistic regression models were constructed to examine associations between adversity and ASD-related service use, accounting for identified covariates. Given the significant amount of missing values of household income, we used six imputed federal poverty level variables provided by the US Census Bureau. Due to the complex sampling design of the survey, the analyses used cluster and sampling weights following the guidance provided by the US Census Bureau with the public use NSCH datasets, in order to adjust for nonresponse and unequal selection bias. 

All data preparation and composite variable construction were completed using R version 6.3.0 (The R Foundation for Statistical Computing) and RStudio version 1.2.5001. Preliminary analyses were conducted in SPSS (v. 27), while STATA (v. 16.1) was used for the logistic regression analyses. Results were considered statistically significant at *p* < 0.05.

These analyses were determined by the University Hospitals Cleveland Medical Center Institutional Review Board to not constitute human subjects’ research.

## 3. Results

### 3.1. Demographics

A total of 114,476 surveys were completed in the period 2016–2019 in which 3462 respondents reported that their child had ever been diagnosed with ASD, and 3247 (weighted population prevalence of 2.9%) had a current ASD diagnosis. After list-wise deletion of missing values across variables, the final analytic sample was 3181 (Table 1). Compared to those without ASD (data not shown), children with a current ASD diagnosis were more often male (79% vs. 50%, *p* < 0.001), older (median 11 vs. 10 years, *p* < 0.001), more often reported inadequate and/or gaps in health insurance coverage (39% vs. 33%, *p* = 0.002), less often received care in a medical home (33% vs. 48%, *p* < 0.001), and more often reported adversity (Any ACEs: 59.6% vs. 34%, *p* < 0.001). Children with ASD were more than twice as likely to have experienced social adversities (15.2% vs. 7.1%, *p* < 0.001), and more than three times as likely to have experienced relational adversities (15.4% vs. 4.8%, *p* < 0.001) than those children without an ASD diagnosis.

### 3.2. Risks and Adversity

Among children ASD, 51% were reported to have mild ASD, 39% moderate, and 10% severe. Although nearly all children with ASD (90.4%) qualified as having complex special health care needs, very few (7.9%) were described by their parent as being in fair or poor overall health. In total, 79% of children with ASD in the analytic dataset had medical health risks, defined as two or more of the following: complex special health care needs, fair or poor overall health, functional limitations, 2 or more total chronic conditions (including ASD). In bivariate analyses, there were no significant differences among children with ASD with and without additional medical risks by child age, race and ethnicity, insurance status, household income, caregiver education, or social risks. Severity of ASD was significantly associated with the presence of two or more medical risks (see Table 2).

The most common social adversities reported by US children with ASD were household economic hardship (finances often not sufficient to cover basics like housing and food—37%) and food insecurity (10%). There were substantial differences in experience of social adversities by child age, ASD severity, race/ethnicity, child sex, caregiver education, and parent centered medical home (see Table 2). Black non-Hispanic children most often reported social adversities (18%), compared to 17% of Hispanic children, 14% of children identifying as multi-racial or other (including Asian, Pacific Islander, American Indian and/or Alaska Native), and 10% of White non-Hispanic children with ASD (data not shown).

Only 40% of US children with ASD reported no exposures to relational adversity (see Table 1). The most common relational adversities were parental divorce (32%) and frequent parental aggravation or anger with the child (32%). Approximately 17% of children with ASD live with an adult with mental health concerns and 13% live with an adult with a history of substance use. More than 14% of parents of children with ASD described themselves as being in poor or fair mental health. However, very few (3.4%) parents indicated that they themselves were not coping well with the demands of parenting. In bivariate analyses (Table 2), there were no differences in report of relational adversities by race/ethnicity, child sex, and insurance coverage, though relational health risks were significantly associated with child age, ASD severity, and patient-centered medical home. 

### 3.3. Receipt of Prescription Medication for ASD

Among all US children with ASD, nearly one in three (29%) reported receiving medications to treat ASD-related symptoms (Table 1). In bivariate analyses (Table 2), receipt of prescription medication for ASD symptoms was significantly associated with child age, ASD severity, race/ethnicity, reports of two or more medical risks, and two or more relational adversities. There were no significant differences in report of medication use to treat ASD-related symptoms by child sex, income, insurance coverage, parent education, care in medical home or experience of social adversities. In multivariable analyses accounting for these factors (Table 3), children reporting two or more relational adversities had significantly greater odds of receiving medication to treat their ASD-related symptoms (OR 1.5, 95%CI: 1.02, 2.18), as did older children (OR 1.15, 95%CI:1.10, 1.19) and those with medical risks (OR 4.27, 95%CI:2.6, 7.01).

### 3.4. Behavioral Therapy for ASD

Only 62% of US children with ASD indicated receiving in the prior year behavioral therapy to address their ASD-related symptoms (Table 1). In bivariate analyses (Table 2), there were no significant associations among use of behavioral therapies and race/ethnicity, child sex, income, insurance coverage, caregiver education, medical home, or experiences of adversity, while child age, ASD severity, and medical risks were significantly associated with receipt of behavioral therapy. Interestingly, in multivariable analyses (Table 3), female sex, younger age, ASD severity, household income, and report of two or more medical risks were all significantly associated with receipt of behavioral therapies. Accounting for these factors as well as insurance status, race/ethnicity, and care in complete medical home, there was no evidence for an association between receipt of behavioral therapy and neither experience of multiple social adversities (OR 0.8, 95%CI 0.49, 1.31), nor experience of multiple relational adversities (OR 1.5, 95%CI: 0.97, 2.31).

## 4. Discussion

In this nationally representative dataset, we find that children with ASD are at greater risk for exposure to adversity than typical peers. Among children with ASD, social and relational adversities are not only prevalent, but may influence use of prescription medications to treat symptoms associated with ASD, independent of other sociodemographic and clinical features that have previously been shown to complicate access to care and timely engagement with needed services. Accounting for key factors predisposing need for and enabling use of health services for ASD, we found that children with ASD who have experienced relational adversities are substantially more likely to receive medications for their ASD than children without those risks. By contrast, neither social nor relational adversity were significantly associated with use of behavioral therapies, all else being equal.

Our findings are consistent with data from earlier iterations of the National Survey of Children’s Health. For example, studies using data from the 2011–2012 NSCH also found that children with ASD were more likely to have experienced ACEs than neurotypical youth [22], and that among children with ASD, experience of 1–2 ACEs and 3 + ACEs was associated with prolonged time to diagnosis [18]. It is possible that ACEs interfere with obtaining care, and/or that behavioral symptoms associated with ACEs complicate ASD presentation. For example, Kerns and colleagues [23] found that the association between ASD and ACEs was stronger for lower income children, though this relationship diminished after controlling for childhood mental health disorders.

Behavior symptoms related to ACEs may also complicate ASD management, as illustrated by our finding of increased odds of use of medications to manage ASD symptoms among children exposed to relational adversities. It is not clear from these analyses whether such medication use represents overuse, underuse, or appropriate treatment based on child symptomatology. The relationships among ASD symptom severity, parent stress and parent mental health and coping are well explicated in the literature [24,25]. Nevertheless, given that the criteria for relational risk includes a parent who is not coping well with the tasks of parenting or who frequently finds themselves aggravated or angry with their child, proactive screening of children with ASD for relational risks may be useful in several ways. For example, such screening may allow health care providers to identify those parents who would benefit from the provision of additional psychosocial supports [26] or enrollment in parenting interventions that would expand their set of tools to manage challenging behaviors [27,28]. This finding is also consistent with emerging literature affirming that safe stable nurturing relationships are essential for promoting resilience and adaptive skills across the lifespan [5].

Notably, we found no evidence for an association between adverse experiences and use of behavioral therapies for ASD. This is in contrast to prior work with earlier versions of this dataset that found with regard to accessing behavioral health treatment, youth with ASD who had 1–2 and 3+, ACEs had a 22% and 27% relative increase in the median age of entry into behavioral therapy services [18]. We speculate that access to behavioral therapies for ASD overall may be more limited than access to medications. For example, there may be substantial geographic variation in the availability of specialists to provide behavioral therapies that these analyses. In this scenario, referral to behavioral therapies might vary based on distribution of specialists, and thus less discretionary than medications, which might be prescribed by either primary care clinicians or specialists. That is, use of behavioral therapies may be more closely aligned with need factors (e.g., ASD severity, medical risks), health systems factors (e.g., availability of behavioral therapies) and families’ ability to pay for such services out of pocket (e.g., income) than other enabling factors (e.g., insurance, social adversities, relational adversities).

It is also notable that we found no association between social adversities (food insecurity, household economic hardship, experience of racism, neighborhood safety) and odds of medication use or behavioral therapies for ASD, despite expectations based on the literature that these factors should influence access to health care services. For example, food insecurity has been shown to be highly associated with increased health care expenditures and family stress [29,30]. Given the intersectionalities between social adversities and other demographic characteristics (e.g., race/ethnicity, household income, and adequacy of health insurance), it may be that it is necessary to consider a child’s profile “in total”, rather than parsing these factors one-by-one. Further work is needed to generate more comprehensive profiles of children with ASD. Nevertheless, findings from this study as well as from previous studies indicate that the effect of even one ACE might be enough to disrupt parents’ abilities to access and coordinate care for their children [18]. Our findings indicate that clinicians should be alert to the predisposing and enabling factors that influence such access to care “beyond the basics” of insurance type.

There are limitations of the present study. This is a cross-sectional study using a population database consisting of only parent-report responses. Therefore, causality cannot be assessed, nor can we make any assertions regarding timing of events, that is, the timing of adverse experiences relative to timing of diagnosis or treatment. Another limitation of this study is that we do not have information regarding the specific types of therapies received, the length of treatment, nor can we assess the extent to which families continue to engage with services. While we were interested in exploring the relationship among adversity and factors influencing access to and use of care among children with ASD, we were also limited in our ability to assess families’ experience of this process. For example, we relied on “complete PCMH” to serve as our proxy measure for a child who is expected to be well-connected to services and thus to experience timely screening and intervention [31]. In the NSCH, the complete PCMH construct relies upon parent report of a usual source of both sick and well care with a clinician who knows the child well, delivers family centered care, supports care coordination and ensures appropriate referrals when needed. Notably, this definition does not distinguish among primary care vs. specialty settings, and thus we are not able to disentangle nuances of these children’s initial experiences of screening for and diagnosis of ASD from their current experiences of care. Future research integrating parent-reported experiences of care with clinician documentation of timing and circumstances of evaluation and engagement with treatment services for ASD would help to resolve these and other unresolved questions, such as the extent to which adversity affects initiation and continuation of different specific therapies.

Nevertheless, the results of this study highlight the importance of understanding adversity experiences among youth with ASD as well as those suspected of having or being evaluated for ASD. We recommend that psychosocial stressors be screened for and assessed at every point of contact with clinicians (that is, in both primary care and specialty settings). Given our findings, such screening should go beyond social needs to include thoughtful screening for relational adversities. These same childhood adversities have been shown to impact overall health and life trajectory in the general population [2]. It remains to be seen how these adversities will impact the mental and physical health of individuals with ASD across the lifespan.

## Figures and Tables

**Table 1:** Population Characteristics for Multivariable Analytic Sample (*n* = 3,181).

	Sample *n* (Weighted %) or Median [IQR]
Child Sex	
Male	2547 (80.1)
Female	634 (19.9)
Race/Ethnicity	
White, Non-Hispanic	2179 (68.5)
Black, Non-Hispanic	228 (7.2)
Hispanic	387 (12.2)
Multi-racial/Other, Non-Hispanic	387 (12.2)
Child age	11 [8,9,10,11,12,13,14]
Severity of ASD	
Mild ASD	1623 (51.0)
Moderate ASD	1235 (38.8)
Severe ASD	323 (10.2)
Health Insurance	
Insurance gaps and/or inadequate coverage	1297 (40.8)
Current consistent and adequate insurance	1884 (59.2)
Household Income	
0–99% FPL	378 (11.9)
100–199% FPL	522 (16.4)
200–399% FPL	930 (29.2)
400% FPL or above	1351 (42.5)
Social Adversities	
Food insecurity	267 (10.3)
Economic hardship (finances often unable to cover basics)	1068 (37.7)
Experienced discrimination based on race	150 (7.4)
Neighborhood is not safe	208 (7.2)
2 or more social adversities	382 (15.2)
Relational Adversities	
Parents divorced	982 (31.5)
Death of a parent or caregiver	123(3.8)
Incarceration of a parent or caregiver	287 (9.5)
Witness to domestic violence	254 (9.2)
Adult in home with mental health disorder	600 (17.4)
Adult in home with substance use disorder	412 (12.7)
Parent frequently angry or aggravated with child	1192 (31.8)
Parent not coping well with demands of parenting	151 (3.4)
Parent in poor/fair mental health	502 (14.1)
Adverse Childhood Experiences (ACEs)	
0 ACEs	1339 (42.1)
1–2 ACEs	1306 (41.1)
3 or more ACEs	536 (16.9)
Two or more medical risks	
Yes	2531 (79.6)
No	650 (20.4)
Two or more relational adversities	
Yes	573 (18.0)
No	2608 (82.0)
Two or more social adversities	
Yes	374 (11.8)
No	2807 (88.2)
Receives behavioral therapy for ASD	
Yes	1954 (61.4)
No	1227 (38.6)
Prescribed medication to treat ASD symptoms	
Yes	932 (29.3)
No	2249 (70.7)

ASD = autism spectrum disorders; FPL = federal poverty level.

**Table 2:** Bivariate Associations Among Predisposing Characteristics, Enabling Factors, and Need Factors and ASD-Related Health Services Use.

		Two or More Medical Risks		Two or More Social Adversities		Two or More Relational Adversities		Prescriptions for ASD		Behavioral Therapy for ASD	
	**df**	t *	***p***	t *	***p***	t *	***p***	t *	***p***	t *	***p***
Child age	3244	1.782	0.074	**2.659**	**0.008**	**1.962**	**0.049**	**6.873**	**<0.001**	**−4.785**	**<0.001**
	**df**	χ^2^ **	***p***	χ^2^ **	***p***	χ^2^ **	***p***	χ^2^ **	***p***	χ^2^ **	***p***
Consistent and adequate insurance	1	10.461	0.187	7.2437	0.291	12.938	0.067	1.4282	0.58	1.2765	0.665
ASD severity	2	**323.78**	**<0.001**	**69.585**	**0.003**	**115.98**	**<0.001**	**101.86**	**<0.001**	**80.242**	**0.001**
Race/ethnicity	3	1.0586	0.976	**57.551**	**0.009**	22.515	0.125	**64.008**	**0.004**	16.285	0.434
Child ex	1	14.47	0.054	**75.593**	**<0.001**	13.254	0.084	6.434	0.223	23.518	0.051
Income	3	6.0621	0.713	33.148	0.072	22.76	0.075	15.877	0.265	22.752	0.198
Caregiver educational attainment	3	41.231	0.142	**123.18**	**<0.001**	13.662	0.425	2.8268	0.962	21.334	0.518
Patient-centered medical home	1	3.1296	0.408	**92.642**	**<0.001**	**31.723**	**0.004**	0.42899	0.752	0.52878	0.777
Two or more medical risks	1			6.151	0.476	**60.48**	**0.001**	**154.66**	**<0.001**	**41.287**	**0.007**
Two or more social adversities	1					**233.71**	**<0.001**	3.7838	0.394	15.296	0.122
Two or more relational adversities	1							**38.394**	**0.001**	12.703	0.099

* Independent-samples *t*-test; ** Pearson’s chi-square with Rao and Scott adjustment. **Bold** denotes statistically significant association *p* < 0.05.

**Table 3:** Multivariable Logistic Regression Predicting Health Service Use among US Children with ASD, 2016–2019.

	Medications to Treat ASD-Related Symptoms			Behavioral Therapies for ASD		
	OR	95%CI		OR	95%CI	
Child sex (*ref = male*)	1.45	[0.96	2.18]	1.62 *	[1.04	2.51]
Child age	1.15 ***	[1.10	1.19]	0.89 ***	[0.86	0.93]
ASD severity (*ref = severe ASD*)						
Mild ASD	0.44 **	[0.27	0.74]	0.31 ***	[0.17	0.56]
Moderate ASD	0.76	[0.46	1.26]	0.45 *	[0.24	0.84]
Continuous health insurance that is adequate to meet needs	1.07	[0.74	1.54]	1.16	[0.82	1.64]
Household income (*ref = 400% FPL or higher*)						
0–99% FPL	0.80	[0.47	1.37]	0.41 ***	[0.25	0.68]
100–199% FPL	1.07	[0.66	1.73]	0.58 *	[0.34	0.96]
200–399% FPL	0.77	[0.48	1.22]	0.53 **	[0.35	0.81]
Race/ethnicity (*ref = Non-Hispanic White*)						
Hispanic	0.58	[0.33	1.01]	0.70	[0.43	1.13]
Non-Hispanic Black	1.22	[0.77	1.94]	0.92	[0.56	1.51]
Multi-racial or Other	0.68	[0.46	1.02]	0.89	[0.59	1.35]
Two or more medical risks	4.27 ***	[2.60	7.01]	1.63 *	[1.08	2.45]
Two or more relational adversities	1.50 *	[1.02	2.18]	1.50	[0.97	2.31]
Two or more social adversities	0.84	[0.53	1.35]	0.80	[0.49	1.31]
Care in complete PCMH (*ref = no*)	0.92	[0.62	1.35]	0.99	[0.68	1.45]

OR = odds ratios; CI = confidence interval. * *p* < 0.05, ** *p* < 0.01, *** *p* < 0.001; ASD-autism spectrum disorder; FPL = federal poverty level; PCMH = patient-centered medical home.

## Data Availability

The National Survey of Children’s Health public use data files may be accessed at https://www.census.gov/programs-surveys/nsch.html.
